# Supplementary material for: Benefit of Atrial Overdrive Pacing in Patients with Sleep Apnea: A Meta-Analysis
Source: J Clin Med. 2021 Sep 9;10(18):4065. doi: 10.3390/jcm10184065 (PMC8464753; doi:10.3390/jcm10184065)
Supplement: Supplementary file 1 [file jcm-10-04065-s001.zip › jcm-1324911-supplementary.pdf]

Online supplement data 1

Data on OVID MEDLINE (575 articles)

('atrial overdrive pacing'/exp OR 'atrial overdrive pacing' OR 'pacing'/exp OR 'pacing') AND ('sleep apnea'/exp OR 'sleep apnea' OR 'sleep'/exp OR 'sleep' OR 'osa')

Data on Cochrane library (25 articles)

"atrial overdrive pacing" AND "sleep apnea"

Data on EMBASE (871 articles)

('atrial overdrive pacing'/exp OR 'atrial overdrive pacing' OR 'pacing'/exp OR 'pacing') AND ('sleep apnea'/exp OR 'sleep apnea' OR 'sleep'/exp OR 'sleep' OR 'osa')
